# Sarcopenia as a prognostic factor for survival in patients with locally advanced gastroesophageal adenocarcinoma

**DOI:** 10.1371/journal.pone.0223613

**Published:** 2019-10-22

**Authors:** Christine Koch, Cornelius Reitz, Teresa Schreckenbach, Katrin Eichler, Natalie Filmann, Salah-Eddin Al-Batran, Thorsten Götze, Stefan Zeuzem, Wolf Otto Bechstein, Thomas Kraus, Jörg Bojunga, Markus Düx, Jörg Trojan, Irina Blumenstein

**Affiliations:** 1 Department of Gastroenterology, Hepatology, Endocrinology and Nutrition, University Hospital, Goethe University, Frankfurt am Main, Germany; 2 Department of General and Visceral Surgery, University Hospital, Goethe University, Frankfurt am Main, Germany; 3 Institute for Diagnostic and Interventional Radiology, University Hospital, Goethe University, Frankfurt am Main, Germany; 4 Institute for Biostatistics and Mathematical Modelling, Goethe University, Frankfurt am Main, Germany; 5 Institute for Clinical Research, (IKF), Hospital Nordwest, Frankfurt am Main, Germany; 6 Central Institute for Radiology and Neuroradiology, Nordwest Hospital, Frankfurt am Main, Germany; 7 Department of General and Viseral Surgery, Hospital Nordwest, Frankfurt am Main, Germany; Universidade do Algarve Departamento de Ciencias Biomedicas e Medicina, PORTUGAL

## Abstract

**Background and aims:**

Patients with gastric cancer often show signs of malnutrition. We sought to evaluate the influence of sarcopenia in patients with locally advanced, not metastasized, gastric or gastro-esophageal junction (GEJ) cancer undergoing curative treatment (perioperative chemotherapy and surgery) on morbidity and mortality in order to identify patients in need for nutritional intervention.

**Patients and methods:**

Two-centre study, conducted in the Frankfurt University Clinic and Krankenhaus Nordwest (Frankfurt) as part of the University Cancer Center Frankfurt (UCT). 47/83 patients were treated in the FLOT trial (NCT01216644). Patients´ charts were reviewed for clinical data. Two consecutive CT scans were retrospectively analyzed to determine the degree of sarcopenia. Survival was calculated using the Kaplan-Meier method, multivariate analysis was performed using the Cox regression.

**Results:**

60 patients (72.3%) were male and 23 (27.7%) female. 45 patients (54.2%) had GEJ type 1–3 and 38 (45.8%) gastric tumors, respectively. Sarcopenic patients were significantly older than non-sarcopenic patients (mean age 65.1 years vs. 59.5 years, p = 0.042), terminated the chemotherapy significantly earlier (50% vs. 22.6%, p = 0.037) and showed higher Clavien-Dindo scores, indicating more severe perioperative complications (score ≥3 43.3 vs. 17.0%, p = 0.019). Sarcopenic patients had a significantly shorter survival than non-sarcopenic patients (139.6 ± 19.5 [95% CI, 101.3–177.9] vs. 206.7 ± 13.8 [95% CI, 179.5–233.8] weeks, p = 0.004). Multivariate Cox regression analysis showed that, besides UICC stage, sarcopenia significantly influenced survival.

**Conclusion:**

Sarcopenia is present in a large proportion of patients with locally advanced gastric or GEJ cancer and significantly influences tolerability of chemotherapy, surgical complications and survival.

## Introduction

Gastric cancer is a potentially curable disease, if it is detected at an early stage and if up-to-date and, if needed, multimodal treatment protocols can be applied [1]. However, survival rates still remain poor even with optimal treatment. Recently, the FLOT protocol was established as the new standard of care in patients with locally advanced, not metastasized gastric or gastro-esophageal junction type I-III (GEJ) cancer, which achieves a 3 year survival of 57% [2,3]. Treatment is often limited by patient- or therapy related factors, notably malnutrition due to a variety of factors such as stenosis, loss of appetite, decreased gastrointestinal motility, mucositis, changes in taste sensation or loss of taste, nausea and vomiting during chemotherapy or a combination thereof [4,5]. Malnutrition is a key driver in the development of sarcopenia, which is defined as a loss of muscle mass and function [6,7] and which is a known risk factor in gastric cancer patients for enhanced toxicities of chemotherapy, perioperative morbidity and mortality as well as survival [8–16]. However, published data so far mainly address surgical outcomes and do only rarely include patients undergoing multimodal treatment as it is the current standard of care in Europe. Since especially patients with gastric cancer are at risk of developing malnutrition and consecutive sarcopenia, we sought to determine the influence of sarcopenia on chemotherapy-associated toxicities as well as perioperative morbidity and mortality in patients treated with perioperative chemotherapy and surgery.

We have collected data on 83 patients with locally advanced, not metastasized gastric or GEJ cancer (cT2/3, cN0-3, cM0) who received perioperative chemotherapy, and analyzed for risk factors, including sarcopenia, influencing their long-term survival.

## Patients and methods

### Patients

Electronic patients´ charts (Orbis, AGFA healthcare, Bonn, Germany, and Gießener Tumordokumentationssystem (GTDS), Gießen, Germany) were retrospectively reviewed for the following items: age, sex, tumor type, histology, TNM stage, UICC stage, treatment, Clavien-Dindo-Score, BMI and survival data. Inclusion criteria were histologically proven adenocarcinoma, perioperative chemotherapy and evaluable CT scans to determine the degree of sarcopenia. The study was conducted in two hospitals (Universitätsklinikum Frankfurt and Krankenhaus Nordwest) that are part of the University Cancer Center Frankfurt (UCT). Written, informed consent was obtained from each patient included in the study. 47/83 of the patients were treated in the FLOT trial (NCT01216644). Permission to use these patients´ data for the present study was kindly granted by the leading investigator (SE-AB). The other patients were treated with perioperative chemotherapy outside a clinical trial. Chemotherapy regimens included FLOT (5-flurouracil, leucovorin, oxaliplatin and docetaxel), EOX (epirubicin, oxaliplatin and capecitabine) and ECX (epirubicin, cisplatin, capecitabine) according to local standards. Surgical resection was performed according to local and international standards, taking tumor localization and individual patient-related factors into account. In patients with gastric cancer, GEJ II and III tumors, subtotal gastrectomy, total gastrectomy and transhiatal extended gastrectomy was performed. In all three procedures, Roux-Y-reconstruction was used to restore the esophageal-enteric continuity. In patients with GEJ I tumors and in a few patients with GEJ II tumors, Ivor-Lewis esophagectomy was performed. Lymphadenectomy was done according to international standards for compartment I and II (D2 lymphadenectomy). In patients with Ivor-Lewis esophagectomy, a standardized 2 field lymphadenectomy was performed. All patients were discussed in the interdisciplinary tumor conference (radiology, oncology, gastroenterology, surgery, radiation therapy, pathology) at diagnosis, before and after surgery. The study was approved by the Ethical Committee at the University Hospital Frankfurt (March 6^th^, 2017) and the Landesärztekammer Hessen (November 24^th^, 2017; project number: MC 191/2017) and conforms to the ethical guidelines of the 1975 Declaration of Helsinki.

### Determination of sarcopenia

The method has in detail been described elsewhere [17]. Shortly, CT scans (66/83 pre-therapy, 17/83 preoperative) were retrospectively analyzed to determine the degree of sarcopenia. For this, mean total muscle area (TMA) was measured at L3 and set in relation to body height, resulting in the skeletal muscle index, SMI. SMI = TMA [cm^2^] / height [m]^2^. Sarcopenia was defined as follows: male patients: BMI (kg/m^2^) <25: SMI <43 cm^2^/m^2^; BMI (kg/m^2^) ≥25: SMI<53 cm^2^/m^2^; female patients: SMI <41 cm^2^/m^2^ (regardless of the BMI). Software: AW VolumeShare 7, GE Healthcare, Little Chalfont, UK. For most analyses, patients were divided in two groups based on the presence or absence of sarcopenia.

### Statistical analysis

Statistical analyses were performed according to international standards and have been described by us and others before [18]; analysis was done using International Business Machines Corporation (IBM) Statistical Package for the Social Sciences (SPSS) for Windows (version 22.0; IBM, Chicago, IL, USA), BiAS (version 11, Frankfurt, Germany), and R (version 3.5.1, Vienna, Austria). Categorical variables were described in frequencies and percentages. Continuous variables were represented as a median and its range. Categorical variables were compared by the chi-squared (χ2)-test or Fisher’s exact test, as appropriate. Continuous variables were compared using the Wilcoxon-Mann-Whitney-U test.

The baseline for follow-up was the date of initial diagnosis, as confirmed by histology. End of follow-up was the date of death, the last date the patient was known to be alive or data closure (March 6^th^, 2017). Factors associated with postoperative complications were assessed by logistic regression analysis.

Survival was estimated using a Kaplan-Meier survival analysis considering the time to death in months. Survival curves were generated using the Kaplan-Meier method and differences in survival were tested with the log-rank test and with univariate and multivariate Cox Proportional Hazards regression analysis. Results were expressed as hazard ratios (HR) with 95% confidence intervals (CI). All tests were two-sided and p-values ≤0.05 were considered statistically significant.

## Results

### Demographics

**Fig 1:**
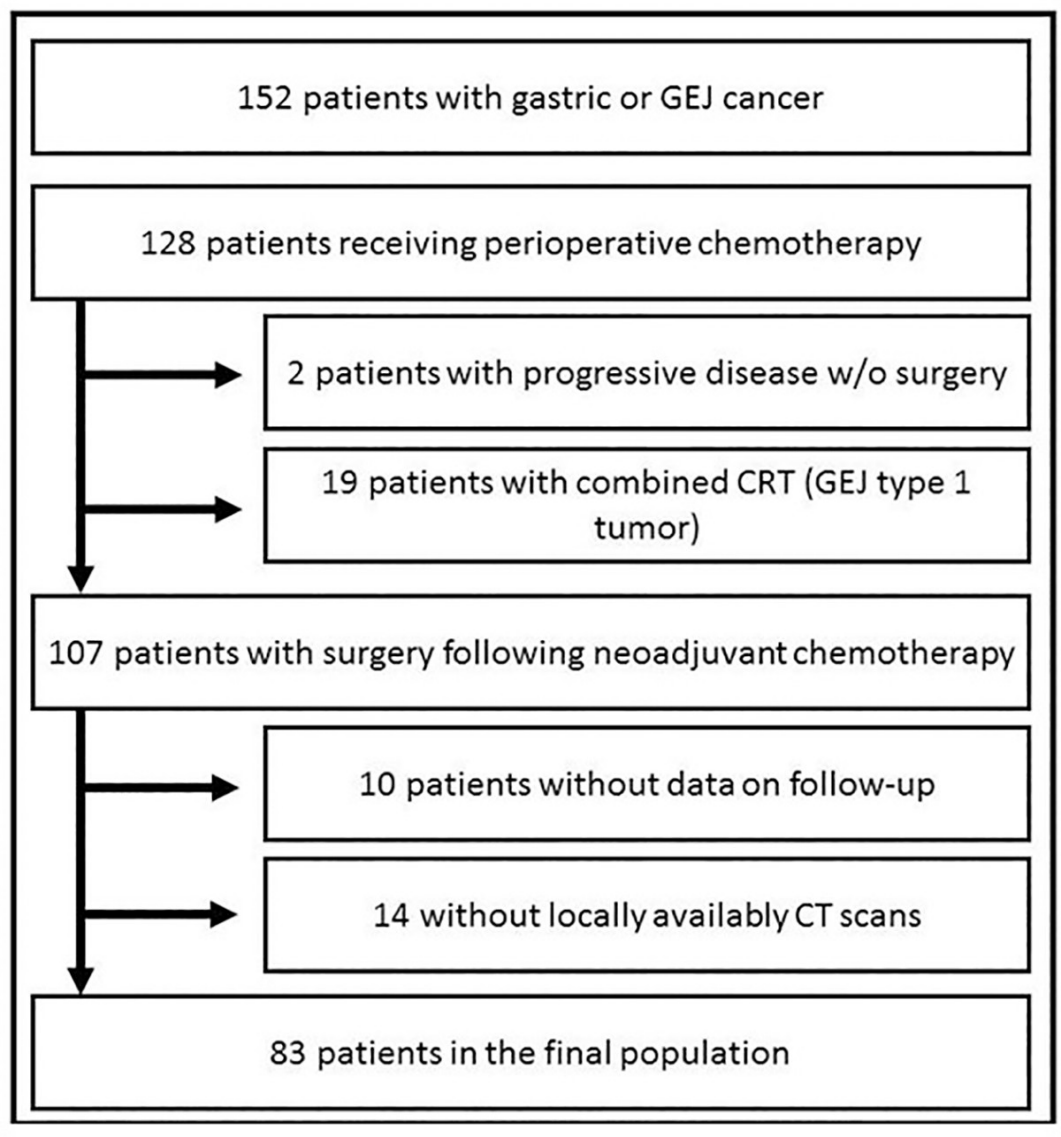
Flow diagram. 60 patients (72.3%) were male and 23 (27.7%) female. 45 patients (54.2%) had GEJ type 1–3 and 38 (45.8%) gastric tumors, respectively. Details on demographic data are given in **Table 1**.

In total, data on 152 patients were reviewed. 83 patients who fulfilled in- and exclusion criteria were included in the study, **Fig 1** shows the study’s flow diagram.

30/83 patients (36.1%) were considered sarcopenic according to the definition lined out in patients and methods (male patients: BMI (kg/m^2^) <25: SMI <43 cm^2^/m^2^; BMI (kg/m^2^) ≥25: SMI<53 cm^2^/m^2^; female patients: SMI <41 cm^2^/m^2^ (regardless of the BMI).

### Sarcopenic vs. non-sarcopenic patients

Mean SMI in sarcopenic patients was 42.0 ± 5.6 cm^2^/m^2^ as compared to 53.9 ± 9.6 cm^2^/m^2^ in non-sarcopenic patients (p<0.001; **Table 1**). Mean weight (72.9 ± 14.9 kg vs. 83.4 ± 21.5 kg; p = 0.057) and BMI (24.4 ± 3.7 kg/m^2^ vs. 27.8 ± 6.8 kg/m^2^; p = 0.054) were not significantly different between sarcopenic and non-sarcopenic patients, respectively. Clinical T- and N stages were distributed evenly between the two groups (p = 0.815 and p = 0.914, respectively, **Table 1**). However, sarcopenic patients were significantly older than non-sarcopenic patients (mean age 65.1 ± 9.8 years vs. 59.5 ± 11.8 years, p = 0.041), terminated the chemotherapy significantly earlier (50% vs. 22.6%, p = 0.037) and showed higher Clavien-Dindo scores, indicating more severe perioperative complications (score ≥3 43.3 vs. 17.0%, p = 0.019, **Table 2**).

**Table 1:** Demographics; sarcopenic vs. non-sarcopenic patients.

	Sarcopenic 30 (36.1%)	Non-sarcopenic 53 (63.9%)	p-value
Sex			**.017**
Female	13 (43.3)	10 (18.9)	
male	17 (56.7)	43 (81.1)	
Age (years)			**.042**
mean	65.1	59.5	
SD	9.8	11.8	
Height (cm)			.510
Mean	172.2	174.3	
SD	11.1	8.8	
Weight (kg)			.057
mean	72.9	83.4	
SD	14.9	21.5	
BMI (kg/m^2^)			.054
Mean	24.4	27.8	
SD	3.7	6.8	
SMI (cm^2^/m^2^)			**< .001**
Mean	42.0	53.9	
SD	5.6	9.6	
Surgical method			.426
gastrectomy	15 (50.0)	32 (60.4)	
transthoracic	7 (23.3)	13 (24.5)	
transhiatal	8 (26.7)	8 (15.1)	
CTx regimen			.711
FLOT	21 (70.0)	35 (66.0)	
EOX/ECX	9 (30.0)	18 (34.0)	
T-Stage			.815
1/2	7 (23.3)	15 (28.3)	
3/4	23 (77.7)	38 (71.7)	
N-Stage			.941
N^+^	19 (63.3)	34 (64.2)	
N^-^	11 (36.7)	19 (35.8)	

**Table 2:** Perioperative complications in sarcopenic vs. non-sarcopenic patients.

	Sarcopenic	Non-sarcopenic	p-value
	30 (36.1%)	53 (63.9%)	
Chemotherapy			.037
as planned	10 (33.3)	26 (49.0)	
dose reduction			
terminated early	5 (16.7) 15 (50.0)	15 (28.3) 12 (22.6)	
Clavien-Dindo-Score			.019
0–2	17 (56.7)	44 (83.0)	
3–5	13 (43.3)	9 (17.0)	

### Survival

**Fig 2:**
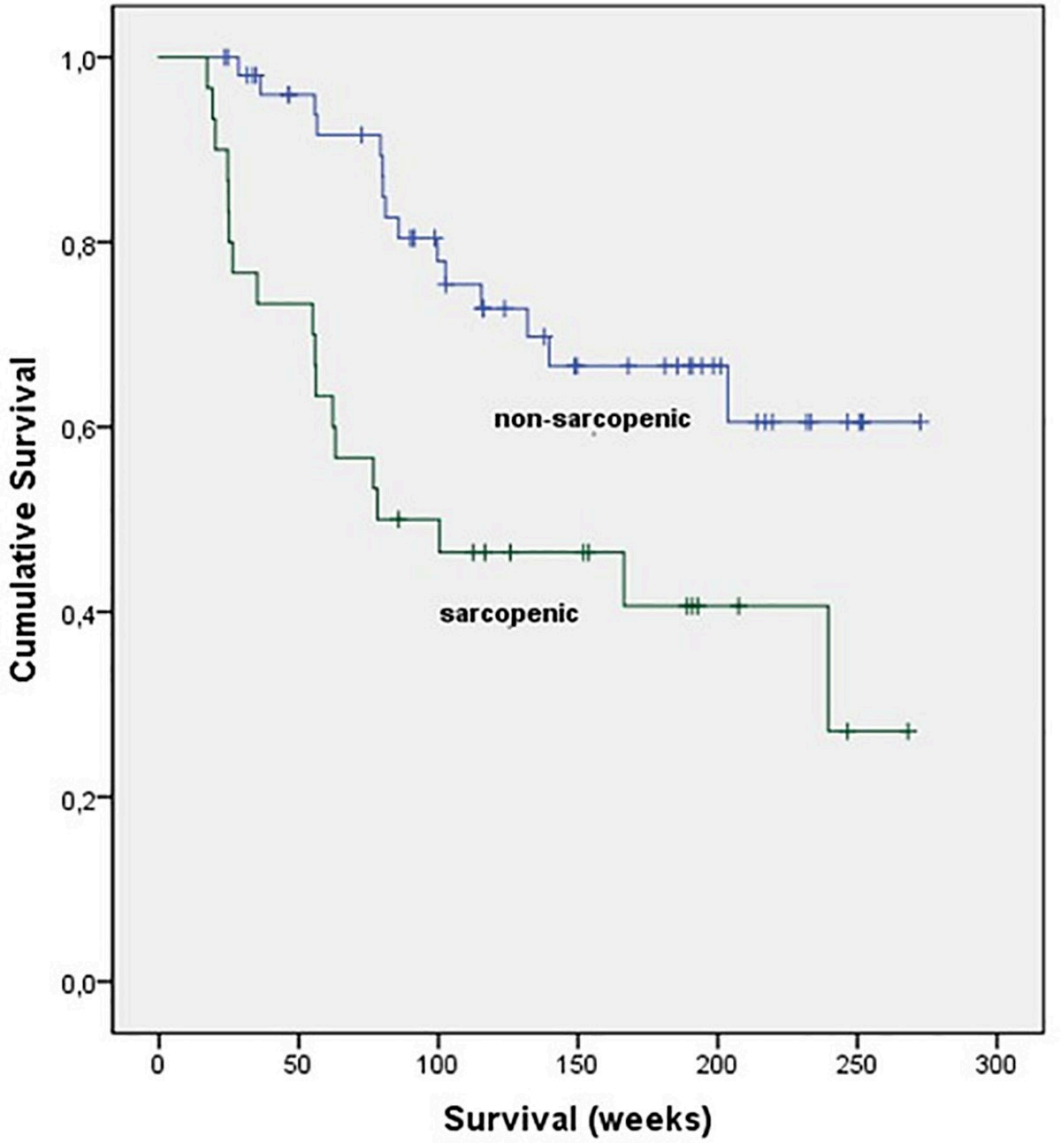
Survival; sarcopenic vs. non-sarcopenic patients; Kaplan-Meier-analysis.

Sarcopenic patients had a significantly shorter median survival than non-sarcopenic patients (139.6 [95% CI, 101.3–177.9] vs. 206.7 [95% CI, 179.5–233.8] weeks, p = 0.004, **Fig 2 and Table 3**).

**Table 3:** Survival; sarcopenic vs. non-sarcopenic patients; Log-Rank test.

Survival, weeks	Median	95% CI lower	95% CI upper	p-Value
Sarcopenic	139.6	101.3	177.9	.004
Non-sarcopenic	206.7	179.5	233.8	

Average time between initial diagnosis and initiation of treatment was 27 days for sarcopenic patients (median 14 days, IQR 34.5) and 23 days for non-sarcopenic patients (median 12.25 days, IQR 33.25, p > 0.2). Since survival in GEJ type I and II patients might be influenced by the surgical method or the kind of anastomosis (transabdominal vs. transthoracic), respectively, we separately analyzed the 36 patients with GEJ type 1 and 2 tumors according to their surgical procedure. Here, we did not find a significant difference in survival if patients were operated with an extended transhiatal resection or a thoracic-transabdominal resection (1 year OS 87.5% vs. 83.9%, 3 years OS 54.8% vs. 57.7%, p>0.2, **Table 4**).

**Table 4:** 1 year and 3 year OS according to surgical method (time-dependent variable).

Surgical method	1 year OS (%)	3 year OS (%)	p-Value
Transthoracic	87.5	54.8	>0.2
Transhiatal	83.9	57.7	

Cox regression analysis revealed that presence of sarcopenia was, besides UICC stage, a risk factor for shorter survival (HR = 2.8; 95%-CI: [1.4;5.7], p = 0.0034). Neither age at diagnosis (p>0.2), sex (p>0.2), localization of the tumor (GEJ tumor vs. gastric cancer, p>0.2), BMI p>0.2) or amount of chemotherapy (as planned vs. dose reduction/terminated early; p>0.2) had a significant influence on survival of the patients in uni- and multivariate analysis (**Tables 5 and 6**).

**Table 5:** Risk factors for survival; univariate Cox regression analysis.

	Hazard ratio	CI 95%	p-Value
age (≤ / > 65) age	0.669 0.999	[0.328;1.372] [0.969;1.030]	>0.2
Sex	1.194	[0.562;2.537]	>0.2
localisation (GEJ vs. gastric tumors)	1.288	[0.599;2.773]	>0.2
BMI (< / ≥ 25)	1.066	[0.531;2.141]	>0.2
BMI	0.978	[0.912;1.040]	
chemotherapy AE (time-dependent variable)	1.303	[0.632;2.687]	>0.2
sarcopenia	2.847	[1.414; 5.731]	0.0034
UICC I / II vs. III	3.459	[1.695; 7.058]	0.0006

**Table 6:** Risk factors for survival; multivariate Cox regression analysis.

	Hazard ratio	CI 95%	p-Value
sarcopenia	4.191	[1.996; 8.799]	0.0002
UICC I / II vs. III	5.002	[2.329; 10.741]	<0.0001

## Discussion

Sarcopenia has emerged as a crucial factor for survival and treatment-related complications in patients with gastric cancer. We evaluated for the first time prevalence and influence on survival in a cohort of patients with locally advanced gastric cancer who underwent perioperative chemotherapy and surgical treatment and determined the presence of sarcopenia during the preoperative period as an important risk factor for survival in these patients. To the best of our knowledge, this is the first report on this finding. Other authors focused on patients undergoing surgery alone [13,16,19–23] or neoadjuvant chemotherapy [15,24] only, which does not reflect the current standard of care in Europe anymore. A recent meta-analysis also focused on surgical outcomes and survival. A significantly poorer survival rate of patients with preoperative sarcopenia was confirmed, however, details regarding perioperative chemotherapy were not reported in this study [25]. We only included patients in our study who underwent multimodal treatment, and sarcopenia emerged as an important predictive factor for survival besides the well-known UICC stage. Bias according to treatment can thus be excluded. Postoperative (and thus also post-neoadjuvant chemotherapy) UICC stage as a prognostic factor for survival in patients with gastric- or GEJ cancer has been widely described [26,27], which was confirmed in our study. This important parameter should be optimized by further refining the neoadjuvant chemotherapy.

We further evaluated dose intensity as a surrogate parameter for chemotherapy-associated toxicities and found that patients with pretherapeutic sarcopenia terminated the chemotherapy significantly earlier and consequently received lower doses of therapy, which might influence the outcome. Tan et al [8] showed in a cohort of 89 patients with esophago-gastric cancer that sarcopenia was a predictive factor for dose-limiting toxicity (DLT). However, DLT was not predictive for shorter overall survival in this cohort, which was confirmed by other authors [15,28] and is in line with our data.

In our cohort, perioperative complications, assessed by Clavien-Dindo score, occurred more frequently in sarcopenic patients. Many other studies confirm this finding [10–13,23,25,29], while others show different results [9].

Importantly, also the pretherapeutic BMI was neither associated with survival nor significantly different in sarcopenic and non-sarcopenic patients, which underscores the need for more sophisticated screening tools than only weight and height. The presence of sarcopenia even in obese patients is a well described phenomenon in oncology [17,30], although this clearly needs more attention in the general practice.

Some authors emphasize the postoperative development of sarcopenia, when comparing pre- and postoperative body composition, as a prognostic factor for poorer overall survival [21,31,32]. We did not perform longitudinal analyses. Instead, and partly in contrast, we found that pretherapeutic sarcopenia already predicts survival, which points to an early nutritional intervention therapy in this cohort of patients.

A limitation of our study is its retrospective design. Furthermore, we detected that our study patients with pretherapeutic sarcopenia were significantly older than non-sarcopenic patients. Sarcopenia was first described more than 25 years ago in elderly patients [33], regardless of the underlying disease, and is a major field of interest in geriatrics and geriatric oncology [6,34]. However, only the presence of sarcopenia and not age at diagnosis was an independent predictive factor for the median survival.

In summary, we were able to identify sarcopenia as an important risk factor for survival in patients with locally advanced gastric cancer. Multimodal treatment of gastric cancer patients has greatly improved overall survival in the past years. However, a further optimization of perioperative chemotherapy and surgical techniques are only two set-screws for further improvement of overall survival and reduction of side effects. Sarcopenia seems to be another crucial factor influencing patients´ outcome. We propose therefore that every patient should be subject to nutritional counselling before starting therapy. The method to determine sarcopenia used in our study is simple, reproducible and does not require additional software or radiological examinations and thus can easily be implemented into clinical routine. Further studies should prospectively evaluate the benefit of individually tailored parenteral nutrition during neoadjuvant treatment in patients with locally advanced gastric cancer.
